# Encouragement of Quackery in Georgia

**Published:** 1852-08

**Authors:** 


### ENCOURAGEMENT OF QUACKERY IN GEORGIA.

The *Southern Medical and Surgical Journal* says: "Georgia is, we believe, the only State in the Union, that has drawn upon its treasury for the encouragement of quackery. The last session of her Legislature appropriated five thousand dollars to the Thompsonian or Botanic School, located at Macon."

This is humiliating. It is a premium to ignorance—an outrage upon science, and, in the estimation of educated men all over the world, disreputable to the Commonwealth. Let quackery have its course; leave it to the people, to adopt or reject it; but for statesmen—framers of laws, to go about patronizing such a system as Botanic Medicine, is simply disgraceful.

